# Analysing child linear growth trajectories among under-5 children in two Nairobi informal settlements

**DOI:** 10.1017/S1368980019000491

**Published:** 2019-04-03

**Authors:** Cheikh Mbacké Faye, Sharon Fonn, Jonathan Levin, Elizabeth Kimani-Murage

**Affiliations:** 1 African Population and Health Research Center, Manga Close, Off Kirawa Road, Kitisuru, PO Box 10787–00100, Nairobi, Kenya; 2 University of the Witwatersrand, School of Public Health, Johannesburg Parktown, South Africa

**Keywords:** Child growth, Nutrition, Stunting, Urban poor, Slums, Sub-Saharan Africa

## Abstract

**Objective:**

We sought to identify factors associated with linear growth among under-5 children in two urban informal settlements in Nairobi.

**Design:**

We used longitudinal data for the period 2007–2012 from under-5 children recruited in the two sites between birth and 23 months and followed up until they reached 5 years of age. We fitted a generalized linear model on height-for-age *Z*-scores using the generalized estimating equations method to model linear growth trajectories among under-5 children. Known for its flexibility, the model provides strong parameter estimates and accounts for correlated observations on the same child.

**Setting:**

Two urban informal settlements in Nairobi, Kenya.

**Participants:**

Under-5 children (*n* 1917) and their mothers (*n* 1679).

**Results:**

The findings show that child weight at birth, exclusive breast-feeding and immunization status were key determinants of linear growth among under-5 children. Additionally, maternal characteristics (mother’s age, marital status) and household-level factors (socio-economic status, size of household) were significantly associated with child linear growth. There were biological differences in linear growth, as female children were more likely to grow faster than males. Finally, the model captured significant household-level effects to investigate further.

**Conclusions:**

Findings from the study point to the need to improve the targeting of child health programmes directed at the urban poor population in Nairobi. Specific modifiable determinants of child linear growth, particularly child weight at birth, exclusive breast-feeding, immunization status and mother’s background characteristics, should be considered when designing interventions aiming at addressing child health inequities in these settings.

Growth trajectories are central to monitoring child health. They provide essential indicators of childhood development and can predict potential determinants of adult health outcomes. Building on the WHO standards for child growth^(^ ^1^ ^)^, a simplified classification of child growth trajectories has established the three following types: standard or normal growth; delayed growth; and rapid growth. Research suggests that children with normal growth trajectories are more likely to have better health outcomes than those with abnormal growth^(^ ^2^ ^–^ ^4^ ^)^. Child growth abnormalities refer to deviations in weight, height or head size, and mostly reflect either growth retardation (delayed growth) when the deviations are below standards or growth acceleration (rapid growth) when the deviations are above standards^(^ ^1^ ^,^ ^5^ ^)^. Rapid growth is associated with health issues later in life such as hypertension and obesity^(^ ^6^ ^,^ ^7^ ^)^, while delayed growth is associated with both short-term and long-term negative effects on child development, including poor cognitive, psychosocial and schooling outcomes^(^ ^8^ ^)^. Likewise, increased child morbidity and mortality have been reported among the consequences of child growth failures^(^ ^3^ ^,^ ^4^ ^,^ ^9^ ^–^ ^11^ ^)^. In the present study we focus on child linear growth, which refers to growth in terms of crude height or height-for-age *Z*-score (HAZ).

Causes of impaired child linear growth are diverse. Among them, poor/inappropriate nutrition (undernutrition or overnutrition), infectious diseases, unfavourable prenatal conditions and genetic disorders are most documented^(^ ^12^ ^,^ ^13^ ^)^. Undernutrition refers to a deficiency in nutrient consumption which increases biological requirements and prevents the body from absorbing the few nutrients consumed^(^ ^14^ ^)^. On the other hand, overnutrition relates to conditions where intake of food is in excess of dietary energy requirements, resulting in overweight and/or obesity^(^ ^15^ ^,^ ^16^ ^)^. Infectious diseases among children, both acute and chronic infections, have a considerable effect on linear growth as they provoke a systemic response and/or affect children over a sustained period^(^ ^17^ ^)^. Among prenatal conditions that are associated with child growth failures, premature birth, low birth weight, and mother’s poor nutrition or diseases such as diabetes and CVD are widely documented^(^ ^18^ ^)^. Finally, genetic disorders with the potential of leading to child growth impairment include pituitary disorders and syndromes such as Turner’s, Noonan’s and Marfan’s, among many others^(^ ^12^ ^,^ ^19^ ^)^. In the present study, we do not focus on genetic causes of child growth impairment.

In African countries, approximately 40 % of children under 5 years of age (under-5s) are affected by growth impairment including linear growth retardation (36 %) or overweight (7 %)^(^ ^8^ ^)^. Child linear growth retardation is more pronounced in sub-Saharan Africa, particularly Eastern and Southern Africa, where the prevalence of stunting reached 40 % among under-5 children according to UNICEF 2007–2011 data^(^ ^20^ ^)^. In Kenya, the prevalence of linear growth failure (stunting) among under-5 children has decreased from 35 % in 2008–2009^(^ ^21^ ^)^ to 26 % in 2014^(^ ^22^ ^)^. The corresponding figure for Nairobi, the capital city of Kenya, was 17 % in 2014^(^ ^22^ ^)^. However, this figure hides intra-urban disparities. Indeed, child stunting was estimated at 46 % in 2010 in two of Nairobi’s informal settlements, Korogocho and Viwandani^(^ ^23^ ^)^, covering a total population of roughly 70 000 residents^(^ ^24^ ^)^.

Despite this, few studies have focused on the determinants of child growth in slums. Children’s attributes at birth (sex and weight) and mothers’ background characteristics, particularly education, marital status and health-seeking behaviour, are associated with abnormal child growth, specifically stunting^(^ ^25^ ^–^ ^28^ ^)^. In these studies, stunting was documented^(^ ^23^ ^,^ ^29^ ^)^, with more emphasis on the burden at a specific period rather than understanding key differences between children. For instance, it is unclear how child nutritional conditions, mother’s background characteristics and family socio-economic status in these settlements influence child growth in the first 5 years of life. Similarly, studies from other settings reveal that child prenatal conditions at birth may lead to abnormal growth curves^(^ ^18^ ^)^. Therefore, it is of interest to analyse child linear growth trajectories among under-5 children in specific urban informal settlements and attempt to understand how they relate to potential key determinants, particularly prenatal conditions, background of mothers, child feeding and health status.

Modelling of human physical growth involves using high-quality longitudinal data and fitting a model that best describes changes in growth measurements of an individual or population over time^(^ ^30^ ^)^. In the present study, we use parametric models on high-quality and stable longitudinal data sets from the Nairobi Urban Health and Demographic Surveillance System (NUHDSS). Specifically, we fitted a generalized linear model on HAZ using a generalized estimating equations (GEE) approach which accounts for correlated responses on the same child and allows for irregularly timed measurements^(^ ^31^ ^)^.

## Methods

### Study settings

The present study used longitudinal data collected between 2007 and 2012 as part of the Maternal and Child Health (MCH) project implemented by the African Population and Health Research Center (APHRC) in two informal settlements in Nairobi: Korogocho and Vivandani. The two study sites are located approximately 10 km from the city centre and about 7 km from each other^(^ ^24^ ^)^. Korogocho has seven villages and a more stable population (less migration), with about a quarter of its resident population aged 12 years or above having been born in this informal settlement^(^ ^24^ ^)^. By contrast, Viwandani has the same number of villages as Korogocho but is a more transient community which attracts a youthful, more educated and highly mobile population seeking job opportunities in the nearby industries^(^ ^24^ ^)^.

The MCH project was nested within the NUHDSS that APHRC has been running since 2002 in the two sites. Every four months, the NUHDSS records demographic events (births, deaths and migration) and information on pregnancy episodes and outcomes. In addition, information on household characteristics (household possessions and amenities; household livelihoods), education, marital status and employment status are collected every twelve months. The MCH project recruited cohorts of mother–child pairs from the NUHDSS and followed them up every four months. A mother–child pair was recruited if the child was born in the informal settlement and was less than 6 months old at the time of recruitment. In addition to the NUHDSS routine data, the MCH project collected data on maternal reproductive health (pregnancy, delivery, antenatal care including mothers’ height and weight, postpartum period, pregnancy and contraception) and health-seeking behaviour, as well as child health (postnatal care, diseases, feeding practices, vaccination and anthropometric measurements)^(^ ^26^ ^,^ ^32^ ^)^.

### Study population

Between 2007 and 2012, the MCH project recruited 8756 children and their mothers through thirteen cohorts. The study sites are characterized by high mobility of residents and high attrition rates in the cohorts over the 5-year period of observation, which was confirmed by our analysis. The overall annual attrition rate in the MCH study was 23 %. The two cohorts with the lowest attrition rates were selected for the present study (13 % in cohort 3 and 15 % in cohort 4). In total, 1917 children were recruited for the two cohorts (948 children in October 2007 for cohort 3 and 969 children in May 2008 for cohort 4). Additionally, these two cohorts provide the biggest sample sizes and have sufficient observations throughout the 5-year observation period (see online supplementary material, Fig. S1) to enable in-depth and statistically valid analyses. Children in the two cohorts were recruited between birth and 6 months, on average, and followed up until the age of 5 years. Each child contributed on average 2·4 years of data and the median number of observations per child was 7. Mothers of at least one living child, and for whom no important information (e.g. child date of birth or mother’s age) was missing or implausible, were included in the study.

### Measures

We used HAZ as the main outcome variable, computed at each visit for each child. In addition, we computed other growth variables such as weight-for-height *Z*-scores (WHZ) and weight-for-age *Z*-scores (WAZ) based on the WHO 2006 standards for children under 5 years^(^ ^1^ ^)^.

In the descriptive analyses, the growth measurements (HAZ, WHZ and WAZ) were used to define stunting, wasting, underweight and overweight status based on sd from the median of the WHO reference^(^ ^33^ ^,^ ^34^ ^)^. For instance, severe stunting refers to HAZ<−3, moderate stunting refers to −3≤HAZ<−2, and no stunting to HAZ≥−2. The same classification was used to define wasting (based on WHZ) and underweight (based on WAZ). Overweight status was considered severe (WHZ≥3), moderate (2<WHZ≤3) or normal weight (−2<WHZ≤2). Observations with implausible HAZ values (absolute values of HAZ>6) were dropped from the analysis.

### Covariates

Guided by the current literature and child health conceptual frameworks^(^ ^35^ ^,^ ^36^ ^)^, we considered feeding practices (exclusive breast-feeding and complementary feeding) and health status (immunization and acute illness) as potential key determinants of child growth. WHO current recommendations on child feeding practices include: (i) initiation of breast-feeding for all newborns within the first hour of life; (ii) exclusive breast-feeding for the first 6 months of life; and (iii) continued breast-feeding for 2 years and beyond with nutritionally appropriate and safe complementary foods introduced at the sixth month^(^ ^37^ ^)^. Full immunization status was defined as receiving all the basic childhood vaccinations as recommended by the WHO by the end of 24 months after birth. We assessed child illness based on symptoms reported by the mother or caregiver over the last two weeks preceding each survey round. Specifically, we recorded information on the five following symptoms: diarrhoea, cough, cough with rapid breathing, fever and convulsion.

Finally, the following variables were also considered among the covariates: mother’s background (age, slum of residence, education, ethnicity, marital status, economic status and parity) and child’s conditions (sex, age, birth weight, place of delivery and type of pregnancy, i.e. singleton or twin). Mother’s economic status was estimated during the survey using principal component analysis based on household assets^(^ ^38^ ^)^.

### Analysis

We first used descriptive analyses to characterize the study participants at the time of recruitment. There were a few missing data, particularly on the child length/height measurements that made impossible to compute the growth measurements (i.e. HAZ, WAZ, WHZ). However, the percentage of missing data on these three variables (as shown in Table 1) remained very small (<1·7 %). For each visit, a full case analysis was carried out and there was no imputation of missing values in the descriptive analyses. Then, after recoding the covariates, we fitted a generalized linear model on HAZ using the GEE method to identify the factors associated with linear growth trajectories among under-5 children. The GEE approach is a population-averaged model which accounts for correlated responses on the same child and allows for irregularly timed measurements and time-varying covariates^(^ ^31^ ^,^ ^39^ ^)^. It also allows a more complete use of the available data and produces reliable parameter estimates^(^ ^40^ ^,^ ^41^ ^)^. Since sex differences among children were already accounted for when computing HAZ, we did not fit the model separately for boys and girls. Children born from a multiple pregnancy were excluded from the multivariate analysis and only one child was considered from each household. Therefore, the individual random effects would also include any household-level effects. There were a few missing observations on some covariates, specifically household socio-economic status (11 %) and household size (12 %), as shown in the online supplementary material, Table S1. Given the small amount of missing observations, we did not use imputation as the missing observations were accounted for in the GEE method.

**Table 1:** Characteristics at recruitment of under-5 children (*n* 1917) from two urban informal settlements in Nairobi, Kenya, 2007–2012

Child characteristic	Korogocho (*n* 907)	Viwandani (*n* 1010)	Total (*n* 1917)
Age (months)			
Mean	5·39	6·09	5·76
sd	4·53	4·55	4·55
Minimum	0·66	0·95	0·66
Maximum	22·77	22·80	22·80
			
	%	%	%
Age group			
<6 months	67·0	59·4	63·0
6–11 months	21·8	28·2	25·2
12–23 months	11·1	12·4	11·8
Sex			
Boy	50·1	51·4	50·8
Girl	49·9	48·6	49·2
Stunting status			
Not stunted	81·2	78·4	79·7
Moderate	9·5	14·2	12·0
Severe	7·1	6·6	6·8
Missing	2·3	0·8	1·5
Wasting status			
Not wasted	89·8	94·7	92·3
Moderate	5·5	3·2	4·3
Severe	2·3	1·3	1·8
Missing	2·4	0·9	1·6
Underweight status			
Not underweight	89·4	91·7	90·6
Moderate	6·2	5·4	5·7
Severe	2·4	2·5	2·5
Missing	2·0	0·5	1·2
Overweight status			
Not overweight	89·4	87·7	88·5
Moderate	5·7	8·5	7·2
Severe	4·9	3·8	4·3
Place of birth			
Health facility	71·0	71·7	71·4
Elsewhere	29·0	28·3	28·6
Child’s weight at birth			
Not weighed at birth/missing	35·5	35·0	35·2
Low weight (<2500 g)	4·9	4·0	4·4
Normal weight (2500–5500 g)	59·7	61·1	60·4
Exclusive breast-feeding up to 6 months			
Yes	3·1	1·2	2·1
No	96·9	98·8	97·9
Full immunization			
Yes	53·3	65·9	59·7
No	46·7	34·1	40·3
Number of illness symptoms in the last two weeks			
0	68·3	63·5	66·0
≥1	31·7	36·5	34·0

## Results

### Characteristics of the children

Table 1 shows characteristics of the children at the time of recruitment. Overall, the median age of the children was 4·3 months while the mean was 5·8 months. Children recruited in Korogocho were younger than those recruited in Viwandani, as 67 % of them were aged below 6 months compared with 59 % respectively. The proportions of boys and girls were similar in Korogocho, while the proportion of boys was higher than that of girls in Viwandani.

Findings from Table 1 show that about 19 % of the children were stunted at the time they were recruited (12 % moderate, 7 % severe). The proportion was higher in Viwandani (21 %) as compared with Korogocho (17 %). With regard to wasting status, about 6 % of the children in the two slums were wasted (2 % severely). The prevalence of wasting was twice as high among children from Korogocho as among those from Viwandani (8 *v*. 4 %, respectively). In the two slums, about 8 % of the children were underweight (Korogocho 9 % *v*. Viwandani 8 %). Surprisingly, about 12 % of the children were overweight when they were recruited in the study.

During the survey, we asked whether the child was born in a health facility or elsewhere. The results indicate that about seven children out of ten were born in health facilities, the percentage being almost the same in the two sites. However, the finding that 30 % of the children were born either at home or in a traditional birth attendant facility suggests poor health-care service use or access for a sizeable proportion of children and their mothers at delivery. For children born in health facilities, the weight at birth was recorded in the survey. According to WHO references for weight at birth^(^ ^1^ ^)^, below 2500 g is considered ‘low weight’, between 2500 and 5500 g is considered ‘normal weight’ and above 5500 g is considered as ‘above normal weight’. Based on that classification, the results show that about 60 % of the children were born with normal weight, the proportion being slightly higher in Viwandani (61 %) than in Korogocho (59 %). Children with low weight at birth represented less than 5 % of the two cohorts (5 % in Korogocho *v*. 4 % in Viwandani). However, for more than a third of the children, particularly those born outside health facilities, the weight was not recorded at birth.

Questions were also asked on breast-feeding, specifically on exclusive breast-feeding from birth to 6 months old, and on the duration of breast-feeding. The results show that only about 2 % of the children were exclusively breast-fed in their first 6 months of life.

Regarding child immunization, we noted that about 60 % of the children were fully immunized by 24 months after birth, with differences between the two sites (53 % in Korogocho *v*. 66 % in Viwandani). On child illness, the results show that about two-thirds of the children did not have any symptoms in the two weeks preceding each survey round. The percentage without reported illness symptoms in Korogocho (68 %) was higher than in Viwandani (64 %).

### Characteristics of the mothers

Understanding mothers’ background characteristics is key to proper interpretation of child health outcomes. Indeed, in a number of public health studies, mothers’ background characteristics, in particular their demographic and socio-economic status, were listed among the social determinants of child health outcomes, including child growth and nutrition^(^ ^35^ ^,^ ^42^ ^,^ ^43^ ^)^. In the present study, we collected demographic and socio-economic information on mothers. Table 2 shows that majority of mothers were of a young age (12 % were teenagers and 46 % were aged between 19 and 24 years).

**Table 2:** Distribution of characteristics (%), at recruitment of the child, of mothers (*n* 1679) from two urban informal settlements in Nairobi, Kenya, 2007–2012

Maternal characteristic	Korogocho (*n* 807)	Viwandani (*n* 872)	Total (*n* 1679)
Age at recruitment of the child			
<18 years	15·6	9·2	12·3
18–24 years	40·1	50·4	45·5
25–34 years	35·9	35·6	35·7
≥35 years	8·5	4·8	6·6
Ethnic group			
Kikuyu	29·4	20·6	24·8
Luhya	19·5	15·7	17·5
Luo	30·1	10·9	20·1
Kamba	4·3	35·8	20·7
Missing	0·4	0·3	0·4
Other	16·4	16·6	16·5
Highest education			
No education/pre-primary	5·6	1·6	3·5
Primary	78·0	64·3	70·8
Secondary or above	16·4	34·1	25·7
Household socio-economic status			
Poorest	79·6	56·5	67·6
Least poor	20·4	43·5	32·5
Marital status			
In union	76·9	89·1	83·2
Formerly married	11·7	4·7	8·1
Never married	11·4	6·2	8·7
Parity (index child included)			
1	31·8	40·1	36·2
2	25·7	29·6	27·8
3	17·3	15·5	16·4
4	10·2	8·3	9·2
≥5	15·0	6·5	10·5

The age structure was similar in the two sites; the proportion of older mothers (35 years or above) was higher in Korogocho (9 %) compared with Viwandani (5 %). With regard to ethnic group, the mothers were mostly composed of Kikuyu (25 %), Kamba (21 %), Luo (20 %) and Luhya (18 %). In Korogocho, Luo mothers were the most dominant group (30 %) while in Viwandani, mothers of the Kamba tribe represented the highest proportion (36 %). Looking at educational level, we noted that about 71 % of the mothers reached primary and 26 % went up to secondary level. The proportion of mothers without formal education was higher in Korogocho (6 %) than in Viwandani (2 %). Findings on socio-economic status reveal that about two-thirds of the mothers were classified in the poorest group, while the remaining were considered as least poor. Poorest mothers were mostly found in Korogocho (80 %) as compared with Viwandani (57 %). With regard to marital status, the overall distribution shows that 83 % were married/in union while 8 % were formerly married/in union and the remaining 9 % had never been married/in union. There were, however, more single mothers in Korogocho (23 %) compared to Viwandani (11 %). Information on the number of children ever born (parity) for each mother was collected. Overall, more than one mother out of three had at least three children ever born.

### Child growth trajectories

**Fig. 1:**
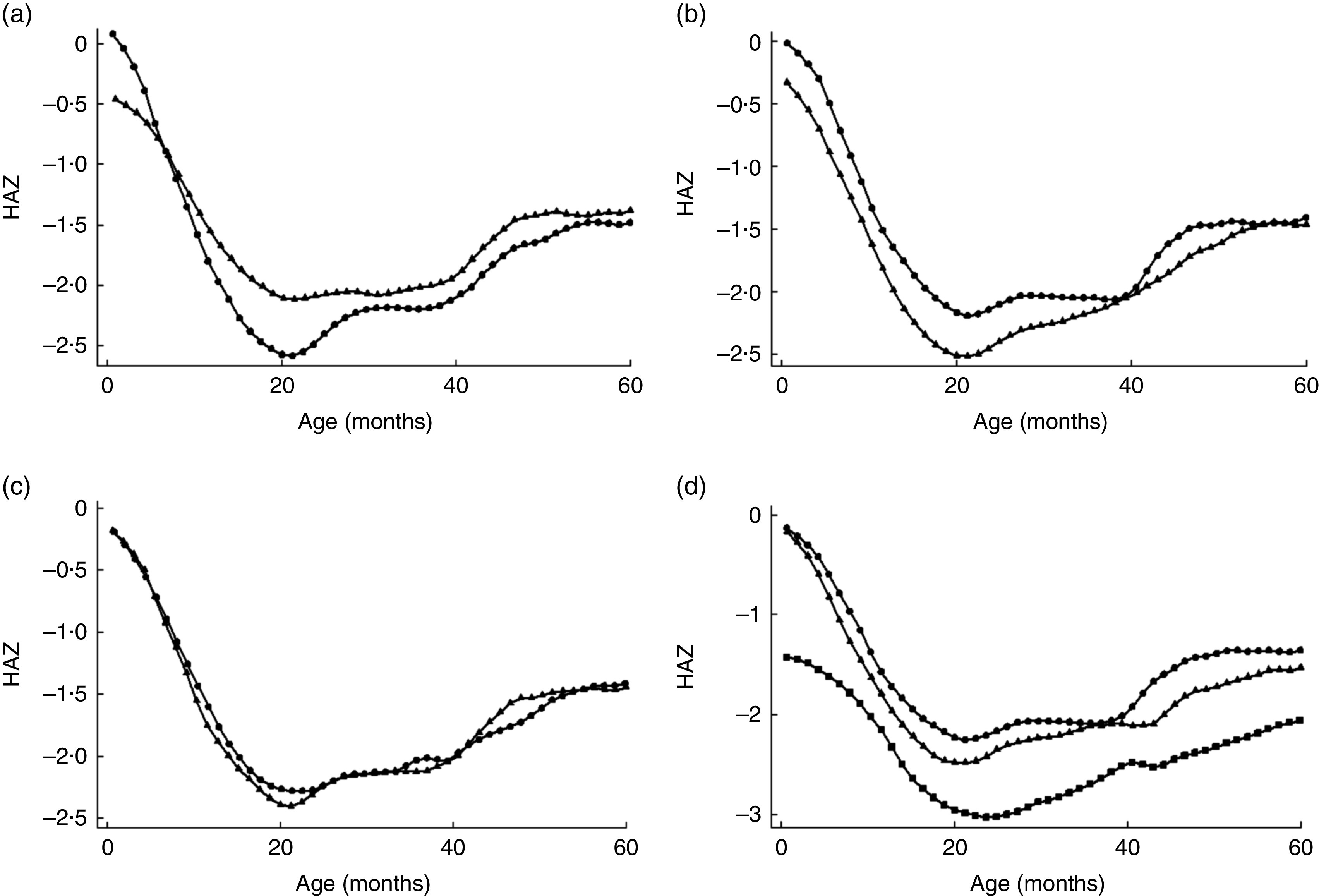
Variation in height-for-age *Z*-score (HAZ) according to specific characteristics: (a) study site (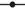, Korogocho; 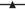, Vivandani); (b) child’s sex (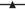, boys; 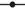, girls), (c) illness symptoms in the last two weeks (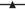, no symptoms; 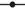, one symptom or more); and (d) child’s weight at birth (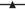, not weighed at birth; 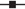, low birth weight; 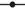, normal birth weight), among under-5 children (*n* 1917) from two urban informal settlements in Nairobi, Kenya, 2007–2012

We plotted children’s HAZ against their age to visualize the growth patterns from birth to 5 years for specific child characteristics. Figure 1 compares child HAZ according to study site, child sex, number of illness symptoms and birth weight.

Overall, we noted that regardless of the characteristic of interest, the HAZ follow a standard pattern with a decline in growth after birth up to about 24 months, then followed by regular catch-up to about 5 years. This growth pattern is unique to developing countries as documented in several studies^(^ ^1^ ^,^ ^27^ ^,^ ^44^ ^)^. However, differences were observed particularly by site, sex and birth weight. Specifically, children living in Korogocho had better linear growth in their first months of life than those in Viwandani; then from about 8 months, the linear growth became greater for children living in Viwandani. Looking at child sex, we noted that girls’ growth pattern remained above that for boys throughout the period 0 to 5 years. We also compared growth patterns by birth weight. As expected, children with low birth weight remained below those with normal birth weight and those whose birth weight was missing. Finally, differences in linear growth were observed between children who had more than one illness symptom over the last two weeks preceding the survey round and those who did not.

### Growth trajectory model

We fitted a fully adjusted growth model for all under-5 children in the two study sites. Findings are presented in Table 3 where a positive coefficient indicates that the variable increases the likelihood of child linear growth.

**Table 3:** Coefficients and 95 % CI associated with determinants of linear growth among under-5 children (*n* 1917) from two urban informal settlements in Nairobi, Kenya, 2007–2012

	All under-5 children		
Covariate	Coefficient	95 % CI	Robust se
Child sex (Ref.=Boy)			
Girl	0·33***	0·19, 0·47	0·072
Site (Ref.=Korogocho)			
Viwandani	0·16(*)	0·00, 0·33	0·084
Place of delivery (Ref.=Health facility)			
Elsewhere	−0·16	−0·43, 0·11	0·139
Weight at birth (Ref.=Normal)			
Not weighed/missing	−0·10	−0·35, 0·14	0·127
Low weight	−0·88***	−1·32, −0·44	0·222
Exclusive breast-feeding up to 6 months (Ref.=No)			
Yes	0·92**	0·28, 1·57	0·328
Full immunization (Ref.=Yes)			
No	−1·30***	−1·43, −1·18	0·064
Illness symptoms (Ref.=No symptom)			
One or more	0·09**	0·03, 0·14	0·029
Mother age group (Ref.=<^18^ years)			
18–24 years	0·23	−0·10, 0·55	0·166
25–34 years	0·37*	0·01, 0·73	0·186
≥35 years	0·28	−0·19, 0·75	0·241
Ethnicity (Ref.=Kikuyu)			
Luhya	−0·07	−0·30, 0·17	0·120
Luo	−0·03	−0·26, 0·20	0·118
Kamba	−0·24(*)	−0·47, 0·00	0·122
Other	−0·16	−0·39, 0·06	0·114
Mother’s education (Ref.=Primary or below)			
Secondary or above	0·09	−0·09, 0·26	0·088
Mother’s marital status (Ref.=Married)			
Formerly married	−0·41***	−0·64, −0·18	0·117
Never married	0·12	−0·16, 0·40	0·145
Mother’s parity (Ref.=^1^)			
2	0·05	−0·14, 0·24	0·098
3	−0·08	−0·33, 0·17	0·127
4	−0·17	−0·47, 0·13	0·153
≥5	−0·10	−0·46, 0·25	0·181
Household economic status (Ref.=Poorest)			
Least poor	0·17**	0·07, 0·28	0·055
Size of household (Ref.=≤^2^)			
3–4	0·47***	0·22, 0·72	0·126
≥5	0·30*	0·02, 0·57	0·140
Constant	−4·02***	−4·45, −3·58	0·220
Ln(child age)	1·00		(Exposure)

Ref., reference category. (*)*P*<0·1, *P<0·05, **P<0·01, ***P<0·001.

Findings in Table 3 identified the key factors associated with linear growth. For instance, area of residence, child-related variables (sex of the child, birth weight, exclusive breast-feeding, immunization status), maternal characteristics (mother’s age, marital status) and household-level factors (economic status, size of household) were significantly associated with child growth. As expected, there were biological differences in linear growth as female children appeared to grow faster than male children. Among other child-related factors, we noted that low birth weight was associated with slower growth while full immunization status was related to faster growth. In addition, children who were exclusively breast-fed in the first 6 months of life presented better linear growth outcomes than those who were not. On maternal factors, we noted that children from older mothers (25–34 years) were more likely to grow faster than those from younger mothers (age below 18 years). With regard to ethnic group, children of Kamba mothers were less likely to grow faster than those of Kikuyu mothers. Higher education level (secondary or above) of the mother seemed to be positively associated with better growth outcomes; however, the association was not statistically significant. Marital status of mothers also influenced child linear growth outcomes. In particular, children from mothers who were formerly married were less likely to grow faster than those of currently married mothers. Looking at household-level factors, the findings show that mother’s socio-economic status was significantly associated with child linear growth. Specifically, children from least poor households were more likely to grow faster than those from the poorest households. We also noted that size of the household influenced child linear growth, with children from larger households (three members or more) having better linear growth outcomes than those from smaller households (fewer than three members). At site level, we noted that children in Viwandani were more likely to grow faster than those in Korogocho.

## Discussion

We used high-quality and stable longitudinal data sets from the NUHDSS to analyse linear growth trajectories among under-5 children in two of Nairobi’s urban informal settlements. We fitted a generalized linear model on HAZ using a GEE approach to account for correlated responses on the same child and to identify the factors associated with the linear growth.

Overall, the findings show that child weight at birth, immunization status and exclusive breast-feeding up to 6 months were key determinants of linear growth among under-5 children in the two urban informal settlements. These results are consistent with findings from other studies, in particular Cameron *et al*.^(^ ^27^ ^)^, who described children born below 2·5 kg as at a higher risk of linear growth failure during their first 5 years of life. Another study in South-East Asia revealed that low birth weight is a predisposing factor to growth attainment in early life^(^ ^28^ ^)^. Even though the regional and national scopes of these studies might alter the comparability of their findings with those of our current study, our results suggest the need to focus on improving the targeting of the urban poor population in child health programmes in Nairobi.

Another finding is that immunization status was positively associated with child linear growth and that about 40 % of the children were not fully immunized. According to WHO, full immunization during childhood is one of the most effective strategies for preventing a number of child diseases and growth impairments^(^ ^45^ ^)^. Children who are not or fully immunized are known to have adverse health outcomes in early life^(^ ^46^ ^)^. Our results highlight the need for increased efforts above what is done so far, particularly beyond the basic promotional immunization campaigns, to reduce child diseases in these rapidly urbanizing settings. Increased awareness of community health volunteers, health professionals and caregivers in the area could also help reduce missed opportunities for timely vaccination.

Our finding that exclusive breast-feeding up to 6 months was associated with better child linear growth confirms results from various child health studies in similar contexts^(^ ^47^ ^–^ ^50^ ^)^. It also extends the general evidence that optimal breast-feeding as recommended by WHO^(^ ^37^ ^)^ has innumerable immediate and long-term positive effects on child health, as detailed by Black *et al.* ^(^ ^8^ ^)^.

The biological differences in linear growth noted were expected as it is widely documented that female children grow faster during early childhood and infancy than male children^(^ ^1^ ^,^ ^51^ ^)^. The finding that children in Viwandani were more likely to grow faster than those in Korogocho might be reflective of the poorer child health outcomes that were generally found in Korogocho, as compared with Viwandani, in studies by Emina *et al.* ^(^ ^24^ ^)^ and Faye *et al.* ^(^ ^52^ ^)^. Similarly, our finding that age of the mother was an important determinant of child linear growth confirms the results from similar studies. In particular, Abuya *et al.* ^(^ ^25^ ^)^ reported a significant effect of mother’s age on child linear growth. Children from young mothers (below 18 years of age) were more likely to be stunted than those of older mothers. This might be explained by the fact that young mothers living in the slums are mostly single and lack resources to cater for their child’s needs including nutrition and health care. A study by Beguy *et al*.^(^ ^53^ ^)^ shows that about 60 % of sexually active female adolescents aged 12–22 years in these settings had at least one child and most of them were single mothers. Marital status of the mother was identified as another factor associated with child linear growth, as children from married mothers were more likely to have better linear growth outcomes than those from non-married mothers. A study from similar settings in West Africa also observed that single parenthood was associated with poor nutritional outcomes among under-5 children^(^ ^54^ ^)^. The finding suggests a protective effect of mother’s marriage or cohabitation.

Another finding is that children from poorest households were more likely to have adverse linear growth than those from least poor households. This finding is also consistent with studies in similar settings^(^ ^25^ ^,^ ^55^ ^)^.

Our result that size of the household was among the factors associated with child linear growth in the two informal settlements points to the fact that extended families might be more protective for the child than smaller families in the two slums. Children living in larger households may receive more attention from family members, particularly when it comes to nutrition and health care, than those in smaller households. Similar results were found in studies from African countries^(^ ^56^ ^,^ ^57^ ^)^. In addition to fixed effects discussed above, the model also captured significant household-level effects. This finding suggests that practices at household level may contribute to driving child linear growth in these settings. Specific family practices or health-seeking behaviour might be among the uncaptured information as evidenced in similar studies^(^ ^58^ ^,^ ^59^ ^)^. Further studies are needed to have a more complete understanding of the underlying factors of child linear growth in these settings.

### Study limitations

We did not find the expected association between illness symptoms and child linear growth. A number of studies in different settings reported that childhood acute and chronic illnesses are highly associated with linear growth in early life^(^ ^8^ ^,^ ^11^ ^,^ ^12^ ^)^. The absence of an association reported in the present study does not necessarily nullify the effect of diseases in child growth; it rather interrogates the timing of the measurement of the illness as captured in the data sets. For instance, illness symptoms (fever, convulsions, diarrhoea, cough, rapid breathing) were captured only over the two weeks preceding each interview, which might have led to underestimation of the effects of illnesses that occurred before.

Similarly, mother’s height, child gestational age and complementary feeding variables were not included in the model as the information was not appropriately collected during the survey. However, variables on child weight at birth and exclusive breast-feeding might have been appropriate proxies to capture the missed information. We also noted a few differences between mothers of children who were lost to follow-up and those of children observed up to 5 years in terms of age group, parity at child birth, household size and ethnic group (see online supplementary material, Table S2). However, there was no significant difference in terms of individual characteristics between children who were lost to follow-up and those observed up to 5 years (Table S3) and we believe that the missing data incurred did not affect our findings. Finally, basic determinants of child health, specifically the political context and physical environment that were not directly studied, might have been captured by the effects of household-level variables, which will be investigated in a further study.

The importance of normal linear growth in early life in developing countries cannot be overemphasized as it has profound effects on child health outcomes. Findings from the present study point to the need to improve the targeting of the urban poor population in child health programmes in Nairobi. They also call for changes in current programmes addressing child undernutrition in Nairobi slums.

The current study identifies important child and maternal factors that are closely related to linear growth among under-5 children in two of Nairobi’s informal settlements. These specific modifiable factors, particularly child weight at birth, immunization status, feeding and mother’s background characteristics, should be considered when designing interventions aimed at addressing child health inequities in these settings. The interventions must also take account of the enabling role of women’s education (secondary or higher) in ensuring good child health outcomes, including normal linear growth. Similarly, targeting single mothers in child health and economic interventions in these areas would contribute to lowering the prevalence of linear growth impairment among under-5 children.

## Supplementary material

For supplementary material accompanying this paper visit https://doi.org/10.1017/S1368980019000491. click here to view supplementary material
