# Supplementary material for: Analysing child linear growth trajectories among under-5 children in two Nairobi informal settlements
Source: Public Health Nutr. 2019 Apr 3;22(11):2001–11. doi: 10.1017/S1368980019000491 (PMC6570617; doi:10.1017/S1368980019000491)
Supplement: Supplementary file 1 [file S1368980019000491sup.zip › S1368980019000491sup001.docx]

**Fig. S1:** Number of children observed at each survey round by cohort
