# Supplementary material for: Analysing child linear growth trajectories among under-5 children in two Nairobi informal settlements
Source: Public Health Nutr. 2019 Apr 3;22(11):2001–11. doi: 10.1017/S1368980019000491 (PMC6570617; doi:10.1017/S1368980019000491)
Supplement: Supplementary file 1 [file S1368980019000491sup.zip › S1368980019000491sup002.docx]

**Table S1. Number and percentage of missing observations per variable included in the multilevel model**

| Variables included in the multilevel model | Number of missing observations | Percentage of missing observations |
| --- | --- | --- |
| Child sex | 0 | 0% |
| Child age | 0 | 0% |
| Site | 0 | 0% |
| Place of delivery | 0 | 0% |
| Weight at birth | 0 | 0% |
| Exclusive breastfeeding up to 6 months | 0 | 0% |
| Immunization | 9 | 0% |
| Illness symptoms | 14 | 0% |
| Mother age group | 397 | 4% |
| Ethnicity | 0 | 0% |
| Mother education | 403 | 4% |
| Mother marital status | 24 | 0% |
| Mother's parity | 16 | 0% |
| Household economic status | 1130 | 11% |
| Size of household | 1169 | 12% |

**Table S2. Comparison between mothers of lost to follow-up children and those of children observed up to 5 years by background characteristics at recruitment**

| **Characteristics of mothers** | **Mothers of children Not observed up to 5 years (N=1082)** | **Mothers of children Observed up to 5 years (N=590)** |
| --- | --- | --- |
| **Age group at recruitment** |  |  |
| <19 | 7.5 | 5.4 |
| 19/24 ** | 54.3 | 44.8 |
| 25/34 | 33.9 | 39.0 |
| 35+ ** | 4.3 | 10.9 |
| **Ethnical group** |  |  |
| Kikuyu | 24.8 | 25.0 |
| Luhya | 18.5 | 15.7 |
| Luo | 20.5 | 19.4 |
| Kamba ** | 23.5 | 15.5 |
| Other ** | 12.4 | 24.0 |
| Missing | 0.4 | 0.3 |
| **Highest education** |  |  |
| Primary and below | 74.7 | 74.9 |
| Secondary+ | 25.3 | 25.1 |
| **Socioeconomic status** |  |  |
| Poorest | 66.4 | 70.1 |
| Least Poor | 33.6 | 29.9 |
| **Household size at recruitment** |  |  |
| <=2 * | 7.6 | 4.0 |
| 3-4 ** | 60.3 | 46.0 |
| 5+ ** | 32.2 | 50.0 |
| **Marital status at child birth** |  |  |
| In union | 84.2 | 81.5 |
| Formerly married | 7.4 | 9.3 |
| Never married | 8.5 | 9.2 |
| **Parity at child birth** |  |  |
| 1 ** | 39.3 | 29.5 |
| 2 | 29.1 | 24.9 |
| 3 | 15.1 | 19.1 |
| 4 | 8.8 | 10.1 |
| 5+ ** | 7.8 | 16.3 |

**p<0.05; **p<0.01; ***p<0.001*

**Table S3. Comparison between lost to follow-up children and those observed up to 5 years by background characteristics at recruitment**

|  | **Not observed up to 5 years (N=1309)** | **Observed up to 5 years (N=608)** |
| --- | --- | --- |
| **Sex** |  |  |
| Boy | 52.1 | 47.9 |
| Girl | 47.9 | 52.1 |
| **Pregnancy type** |  |  |
| Multiple | 3.6 | 3.8 |
| Single | 96.4 | 96.2 |
| **Stunting status** |  |  |
| Not stunted ** | 77.9 | 83.6 |
| Moderate ** | 13.4 | 8.9 |
| Severe | 7.4 | 5.6 |
| Missing | 1.3 | 2.0 |
| **Wasting status** |  |  |
| Not wasted | 93.0 | 91.0 |
| Moderate | 3.7 | 5.4 |
| Severe | 1.9 | 1.5 |
| Missing | 1.4 | 2.1 |
| **Underweight status** |  |  |
| Not underweight | 90.2 | 91.6 |
| Moderate | 6.1 | 4.9 |
| Severe | 2.7 | 2.0 |
| Missing | 1.1 | 1.5 |
| **Overweight status** |  |  |
| Not overweight | 88.2 | 89.1 |
| Moderate | 7.9 | 5.8 |
| Severe | 3.9 | 5.1 |
| **Place of birth** |  |  |
| Health Facility | 70.1 | 74.2 |
| Elsewhere | 30.0 | 25.8 |
| **Child weight at birth** |  |  |
| Not weighted at birth/missing | 36.6 | 32.2 |
| Low weight (<2500g) | 4.1 | 5.1 |
| Normal weight (2500g-5500g) | 59.4 | 62.7 |
| **Exclusive breastfeeding up to six months** | |  |
| Yes | 2.2 | 1.9 |
| No | 97.8 | 98.1 |
| **Immunization** |  |  |
| Up to date | 60.5 | 58.3 |
| Not up to date | 39.5 | 41.7 |
| **Number of illness symptoms in last two weeks** | | |
| 0 * | 62.8 | 69.0 |
| 1+ | 37.2 | 31.0 |

**p<0.05; **p<0.01; ***p<0.001*
